# Punching Up in Psychiatric Care: Interprofessional Stigma in Borderline Personality Disorder Care Through the Lens of Clinical Social Workers in Ontario, Canada

**DOI:** 10.1192/j.eurpsy.2026.11245

**Published:** 2026-07-31

**Authors:** A. Ahluwalia-Cameron

**Affiliations:** Social Work, University of Windsor, Windsor, Canada

## Abstract

**Introduction:**

Borderline Personality Disorder (BPD) remains among the most stigmatized mental health diagnoses. While provider-based stigma is well documented, less attention has been paid to how stigma manifests across interprofessional teams. In healthcare settings where collaboration is essential, social workers often resist and reproduce stigma while navigating hierarchical dynamics. Understanding these interprofessional tensions is critical to improving care for people living with BPD (PLBPD).

**Objectives:**

This sub-study, part of a larger qualitative project, explores how clinical social workers perceive and experience interprofessional stigma toward PLBPD in Ontario mental health settings. The goal is to provide a concrete example of how stigma is enacted, resisted, and negotiated across professional boundaries. By examining these dynamics, the study highlights how stigma is not only an individual attitude but a relational and structural process. In doing so, it offers a learning case for interprofessional teams, pointing to opportunities for reflection, dialogue, and systemic change.

**Methods:**

Qualitative semi-structured interviews were conducted with 41- social workers in Ontario, Canada between April 2020, and January 2021. Recruitment occurred using professional email lists and snowball sampling. Interviews were conducted remotely (average length 60 minutes), audio recorded and transcribed verbatim. NVivo 12 was used for coding. Major themes were identified using a Critical Realist Analysis. Standard ethics protocols were followed; participants were provided an honorarium.

**Results:**

Participants described pervasive stigma toward PLBPD among nurses, physicians, psychiatrists, and psychologists. Reports included dismissive attitudes, diagnostic invalidation, and discriminatory practices, especially in emergency and inpatient contexts. Many social workers portrayed themselves as advocates or “underdogs,” engaging in “punching up” by critiquing more powerful professions while downplaying their own role in reproducing stigma. Despite acknowledging limited training in BPD care, few recognized personal biases unless directly prompted. These findings suggest stigma is structured not only by individual attitudes but also by professional roles, power asymmetries, and systemic norms.

**Conclusions:**

Interprofessional stigma is a significant yet under-recognized barrier to effective BPD care. Addressing it requires more than provider education; it demands interprofessional training, clear role delineation, and systemic strategies that embed accountability, trauma-informed practice, and team-based destigmatization. Social workers are uniquely positioned to lead these efforts, while also critically reflecting on their own contributions to stigma.

**Disclosure of Interest:**

None Declared

